# The Genomic Organization of the *LILR* Region Remained Largely Conserved Throughout Primate Evolution: Implications for Health And Disease

**DOI:** 10.3389/fimmu.2021.716289

**Published:** 2021-10-19

**Authors:** Lisanne Storm, Jesse Bruijnesteijn, Natasja G. de Groot, Ronald E. Bontrop

**Affiliations:** ^1^ Comparative Genetics and Refinement, Biomedical Primate Research Centre, Rijswijk, Netherlands; ^2^ Theoretical Biology and Bioinformatics, Utrecht University, Utrecht, Netherlands

**Keywords:** leukocyte receptor complex (LRC), leukocyte immunoglobulin-like receptor (LILR), killer immunoglobulin-like receptor (KIR), immunoglobulin Ig-like superfamily (IgSF), non-human primates (NHP), human

## Abstract

The genes of the leukocyte immunoglobulin-like receptor (LILR) family map to the leukocyte receptor complex (LRC) on chromosome 19, and consist of both activating and inhibiting entities. These receptors are often involved in regulating immune responses, and are considered to play a role in health and disease. The human *LILR* region and evolutionary equivalents in some rodent and bird species have been thoroughly characterized. In non-human primates, the *LILR* region is annotated, but a thorough comparison between humans and non-human primates has not yet been documented. Therefore, it was decided to undertake a comprehensive comparison of the human and non-human primate *LILR* region at the genomic level. During primate evolution the organization of the *LILR* region remained largely conserved. One major exception, however, is provided by the common marmoset, a New World monkey species, which seems to feature a substantial contraction of the number of *LILR* genes in both the centromeric and the telomeric region. Furthermore, genomic analysis revealed that the killer-cell immunoglobulin-like receptor gene *KIR3DX1*, which maps in the *LILR* region, features one copy in humans and great ape species. A second copy, which might have been introduced by a duplication event, was observed in the lesser apes, and in Old and New World monkey species. The highly conserved gene organization allowed us to standardize the *LILR* gene nomenclature for non-human primate species, and implies that most of the receptors encoded by these genes likely fulfill highly preserved functions.

## Introduction

**Figure 1:**
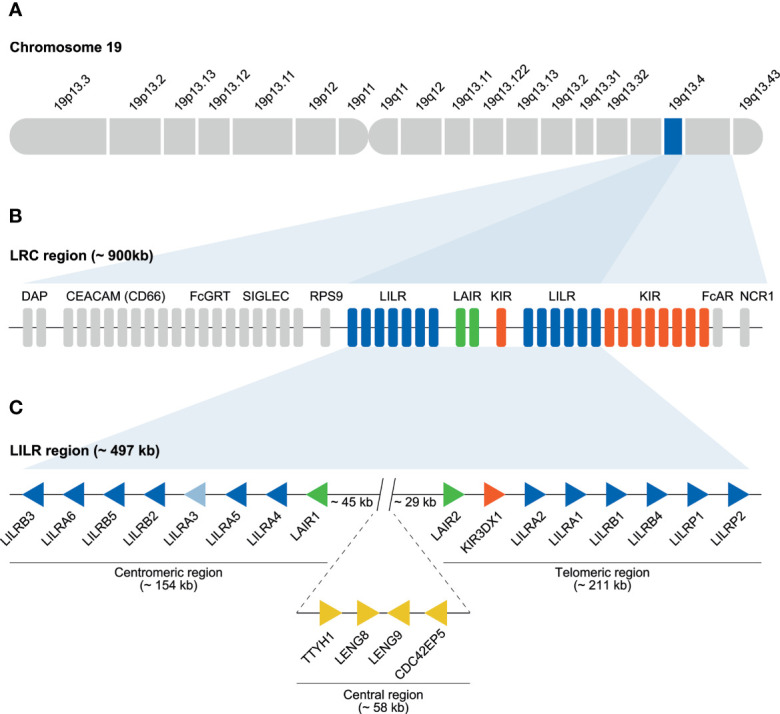
Human LRC region and genomic organization of the *LILR* gene family. **(A)** The LRC region located on chromosome 19q13.4. **(B)** *LILR* (blue bars), *KIR* (red bars), and *LAIR* (green bars) gene families located within the LRC. The remaining genes located in the LRC are indicated by grey bars. **(C)** The *LILR* region (~ 497 kb), schematic illustration of the division of the 11 functional *LILR* genes on the centromeric (~154 kb) and telomeric side of the region (~211 kb) as well as the pseudogenes *LILRP1* and *LILRP2* located on the telomeric side. *LILRA3* is light blue to indicate the *null* haplotype observed in humans. The regions are separated by a central region of approximately 58 kb that includes the genes *TTYH1*, *LENG8*, *LENG9*, and *CDC42EP5*. Between the centromeric and central region, and the central and telomeric region, a stretch of 45 and 29 kb, respectively, is observed. Representing different genes, the arrows are aligned in such a way that they point in the direction of transcription.

The human immunoglobulin superfamily (IgSF) represents more than 700 cell-surface and secreted receptors, which are characterized by the presence of one or more immunoglobulin-like (Ig) domains (1). Several IgSF subfamilies are encoded within the leukocyte receptor complex (LRC), which spans approximately 900 kb on chromosome 19 (**Figure 1A**) (2). This complex encodes the leukocyte immunoglobulin-like receptors (LILR), the killer immunoglobulin-like receptors (KIR) and the leukocyte-associated immunoglobulin-like receptors (LAIR) (**Figure 1B**) (3–5). Other immune-related genes embedded in the LRC are those encoding for the natural cytotoxicity receptor 1 (NCR1) and the Fc-alpha receptor (FcAR) (2, 6). The extended LRC region is located centromeric of the LRC, and was formed by multiple duplication events, eventually resulting in the formation of additional gene families, including sialic acid-binding immunoglobulin-type lectins (SIGLEC), neonatal Fc receptor (FcGRT), the carcinoembryonic antigen-related cell adhesion molecule (CEACAM/CD66), and pregnancy-specific glycoprotein (PSG) (6). Although the extended LRC region encompasses multiple IgSF subfamilies, only the genomic organization of the LRC region, encoding the *LILR*, *KIR*, and *LAIR* gene families, has been reviewed on a regular basis (2, 6, 7).

The *KIR* region, located on the telomeric side of the *LILR* gene cluster, is known to be highly dynamic, and, at population level, haplotypes may show considerable diversity in gene architecture and allelic content (5, 8, 9). The diversity of the *KIR* region is a result of substantial homologous recombination and unequal crossing-over events (10–13). The KIR receptors are expressed by NK cells and subsets of T lymphocytes, and play a key role in immune regulation by interacting with polymorphic epitopes on major histocompatibility complex (MHC) class I molecules, designated in humans as human leukocyte antigen (HLA) (14–17). Furthermore, KIR receptors play a pivotal role in the recognition and elimination of cells lacking the expression of MHC class I molecules (18, 19).

The *LAIR* gene family consists of two genes that encode a cell-surface (LAIR1) and a soluble (LAIR2) receptor, and are located in the center of the *LILR* region (6, 20, 21). The expression of LAIR is broadly confined to peripheral blood lymphocytes, including NK cells, T and B lymphocytes, neutrophils, monocytes, and macrophages (21–25). LAIR1 and LAIR2 gene products are both collagen-binding receptors, and play a key role in controlling tissue inflammation (24, 26–29).

In contrary to the *KIR* gene family, the organization of the *LILR* gene content is conserved in humans (9, 30). A conventional *LILR* haplotype contains 13 genes, 11 of which encode a functional protein, and two are classified as pseudogenes (**Figure 1C**) (31). The human *LILRP1* gene has an apparent 5’ acceptor splice site in front of the exon encoding the third Ig-like domain, resulting in a pseudo-exon, while *LILRP2* became inactivated due to a 7 bp insert as evidenced of a tandem repeat in the exon encoding the second Ig-like domain (32).

*LILR* gene products are widely expressed by immune cell populations of both myeloid and lymphoid lineages, and several members interact with HLA class I molecules (33–35). In contrast to the KIR receptors, LILR receptors do not interact with polymorphic epitopes on the alpha 1 and 2 domains of HLA molecules, but engage with conserved epitopes on the alpha 3 domain and/or with the highly conserved β2-microglobulin structure (a component of the MHC class I dimer) (33). The *KIR* region has been extensively studied in different primate species, including humans. The *LILR* region, on the other hand, has only been thoroughly studied in humans, and its equivalents in mice, chicken, and other vertebrates such as cattle and pigs (36–43). In this communication, we aim to provide a comprehensive overview of the *LILR* region in humans and of the available genomic data in different non-human primate species.

## The Emergence of LILR-Like Receptors

**Figure 2:**
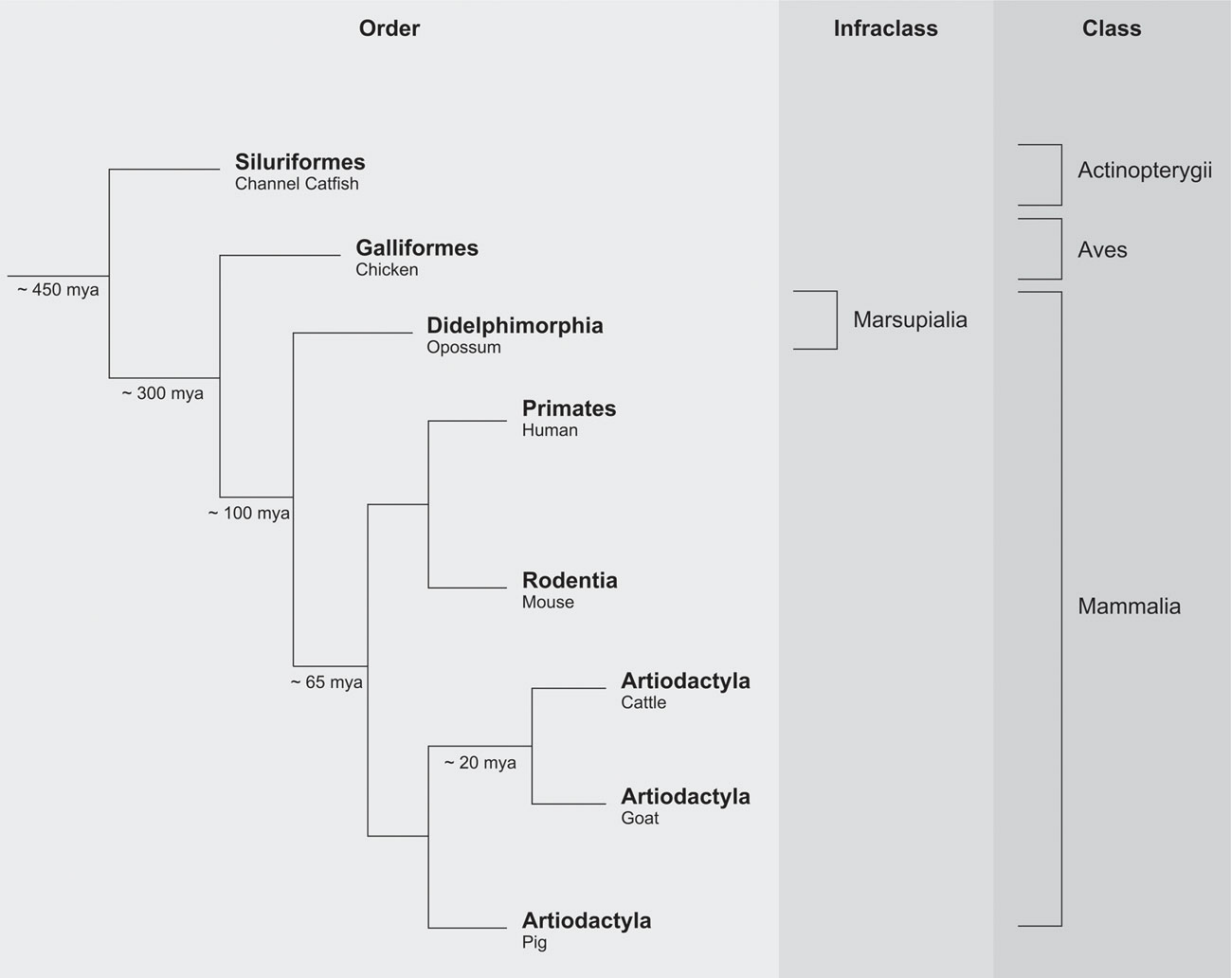
Simplified phylogenetic scheme of vertebrate evolution.

Information regarding the ancestry of any genetic system can be recovered by comparing its presence or absence in different indicator species that once shared common ancestors. Approximately 65 million years ago (mya), the first ancestral mammalian species started to roam the earth. As mentioned previously, the presence of a *LILR* system has been documented in humans and several other species, including mouse and cattle. These data suggest that the emergence of the *LILR* cluster predates radiation of the mammalian lineage (**Figure 2**) (44).

Paired Ig-like receptors (PIR) are encountered in mice, and the genomic architecture of the *PIR* region is comparable to the *LILR* region in humans (39, 45). The order Artiodactyla, which includes cattle, goat, and pig, emerged approximately 65 mya, with cattle and goat diversifying approximately 20 mya. The *LILR* region in pigs turned out to be similar to the human equivalent, but the region itself shows an expansion of the *LILR* genes in the centromeric region (41). In goats, the *LILR* region exhibits a contraction in the number of genes as compared to its human counterpart (42–44). These observations highlight not only that the *LILR* system is subject to purifying selection but also that specialization may have emerged during vertebrate evolution.

The marsupials radiated approximately 100 mya and is represented here by the opossum (**Figure 2**). In opossums, 124 Ig-like domains with similarity to *KIR* and *LILR* Ig-like domains were identified (46). The avian lineage, which emerged approximately 300 mya, provided the next major informative event (**Figure 2**) (44). The chicken immunoglobulin-like receptor (CHIR) gene system is characterized by massive expansion and diversification in comparison to the human *LILR* region. Nonetheless, the highly similar structures found in both humans and chicken suggest that the emergence of the *LILR* cluster might date from before the avian lineage (40, 47, 48). The genes encoding for CHIR and marsupial Ig-like domains have, however, a different transcriptional direction as compared to their evolutionary equivalents in mammalian species.

Data obtained from the class of ray-finned fish (Actinopterygii) evidenced that the origin of the *LILR* system might even date back to approximately 450 mya (**Figure 2**). Ray-finned fish comprise the largest class of vertebrates (~25000 marine and fresh-water species) including, for instance, channel catfish (*Ictalurus punctatus*) (44). In this species, leukocyte immune-type receptors (LITR) have been identified, and have an evident orthologous relationship to human LILR receptors (49).

In summary, there is compelling evidence that a *LILR*-like system, in a way similar to that of the *MHC* complex, emerged before the major expansion of the vertebrate lineage, approximately 450 mya. Some of the LILR receptors have MHC class I molecules as their ligands (**Table S1**), and it is tempting to speculate that both systems co-evolved and had an impact on each other. During vertebrate evolution, the *LILR* complex was subjected to a modest number of expansions and contractions. Some of the receptors may have experienced purifying selection, and therefore still interact with their original ligands. Alternatively, certain LILR receptors of different species may have diverged and specialized, and thereby acquired novel functions. Aside from that, pathogens may have evolved strategies to misuse these types of receptors: for instance, to invade the cell or escape the immune system. An ancestral *KIR* gene existed approximately 50-100 mya, but the primate *KIR* gene cluster arose approximately 30-45 mya (3, 5). The most parsimonious explanation is that initially the presence/absence of MHC class I was scanned by LILR receptors that can diagnose the presence of conserved epitopes. Later, when the *MHC* complex expanded and staged extensive allelic polymorphism, more sophisticated systems – like the *KIR* gene system – arose, which are able to scan for the presence of polymorphic epitopes on MHC class I molecules.

## Genomic Architecture of the *LILR* region And Its Evolution in Humans

Several receptors encoded within the LRC region, including FcAR, NCR1, and various LILR and KIR receptors, function as activating receptors (34, 50–52). The complexity of the immune response and the need to control dangerous immune reactions probably drove the onset of a large arsenal of inhibiting receptors.

**Figure 3:**
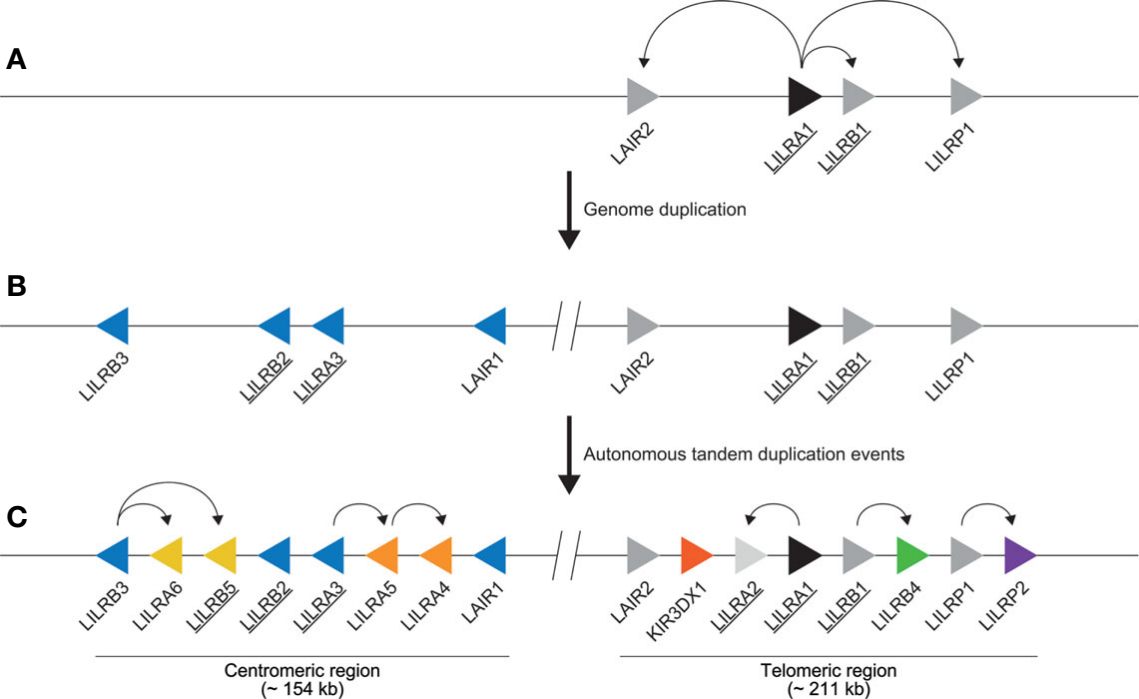
Schematic illustration of the evolution of the human *LILR* region. **(A)** As indicated by the curved arrows, the first primitive *LILR* region arose due to independent *LILRA1* duplication events, resulting in the emergence of *LAIR2*, *LILRB1*, and *LILRP1* (depicted by grey arrows). *LILRA1* is denoted as the ancestral *LILR* gene and is therefore depicted by a black arrow. Genes encoding for receptors interacting with HLA epitopes are underlined. **(B)** The primitive *LILR* region was subsequently duplicated and reversely incorporated into the genome, shaping the centromeric region containing *LILRB3*, *LILRB2*, *LILRA3*, and *LAIR1*, depicted by blue arrows. **(C)** Next, several independent duplication events occurred in both regions, as indicated by the curved arrows, modeling both a centromeric and a telomeric make-up. Representing the different genes, the arrows are aligned in such a way that they point in the direction of transcription. The colored arrows highlight the different autonomous tandem duplication events. The location of *KIR3DX1* gene is indicated (red arrow). The interception with two vertical lines indicates the central region.

It has been proposed that an ancestral gene encoding Ig domains acted as progenitor for *LILR, KIR*, and other immune-related genes such as *FcAR* and *NCR1* (2). This progenitor gene presumably encoded an activating receptor, as it likely featured a positively charged arginine residue in the transmembrane region, because the evolutionarily old *FCAR*, *NCR1*, and *LILR* genes share this feature. The progenitor of *LILRA1* may have acted as the founder gene of the *LILR* cluster (2, 53, 54). Several *LILRA1* duplication events shaped the first primitive *LILR* organization, which contained *LAIR2*, *LILRA1*, *LILRB1*, and *LILRP1* (**Figure 3A**). This event was followed by a relatively stable period, after which time the region duplicated entirely, and was reversely incorporated into the genome, forming the centromeric *LILR* region. The genes in this region are *LILRB3*, *LILRB2*, *LILRA3*, and *LAIR1* (**Figure 3B**). Several autonomous tandem duplication events occurred within the centromeric and telomeric region, and gave rise to the contemporary organization of the *LILR* region in humans, as illustrated in **Figure 3C** (2, 9, 31). The human *LILR* region (~ 497 kb) is divided into the centromeric region (~ 154 kb) and the telomeric region (~ 211 kb). Notably, *KIR3DX1*, previously known as *KIR3DL0* or *LENG12*, is embedded between *LAIR2* and *LILRA2* in the telomeric region (**Figure 3C**). It represents an independent ancestral and highly conserved *KIR* lineage with orthologs in human, chimpanzee, gorilla, rhesus monkey, and common marmoset (55). As the *KIR* gene family arose later in evolution than the *LILR* gene family, it is likely that the *KIR3DX1* gene became inserted into the telomeric section after duplication of the entire *LILR* region.

In humans, most gene products of the telomeric *LILR* region interact with HLA class I ligands, except for receptor LILRB4. Gene products of the centromeric region, however, may dock on HLA and non-HLA molecules (**Figure 3C**) (35). This suggests that the LILR receptors encoded in the telomeric region maintained more or less the original functions, whereas receptors encoded by genes in the centromeric region further specialized and acquired novel functions. Receptor LILRB2, for example, mapping in the centromeric region, mainly docks on HLA molecules but may also interact with angiopoietin-like (ANGPTL) molecules, β-amyloid, and Nogo-66, suggesting that this receptor gained new functions as well (35, 56–59). The genomic architecture of the *LILR* region in humans appears to be relatively stable, and the most common haplotype organization found world-wide is as presented in **Figure 3C**. However, a haplotype with a relative high frequency lacking *LILRA3* as result of a 6.7 kb deletion is also known (referred to as a *null* haplotype) (60–63). The centromeric *LILR* region has an average gene distance of 18 kb (8 kb – 27 kb) between the stop codon and the start codon of adjacent genes, while the telomeric region has an average gene distance of 23 kb (9 kb – 30 kb) (**Figure S1A**). When the UTR regions are included, the centromeric region has an average gene distance of 16 kb (7 kb – 26 kb), while the telomeric region has an average gene distance of 18 kb (9 kb – 27 kb) (**Figure S1B**). Some *LILR* genes have long intergenic regions: for example, the stretch between *LILRB1* and *LILRB4*. Despite these relatively long intergenic regions, no recombination events in the *LILR* region have been recorded (3, 7). This is in sharp contrast to the situation for the neighboring *KIR* region, which is characterized as being highly dynamic and with variable gene numbers and gene combinations, and consists of highly polymorphic genes (5, 12, 64).

## *LILR* Gene Nomenclature in Humans and Non-Human Primates

In the past, the terms Ig-like transcripts (ILT), leukocyte Ig-like receptors (LIR), and CD85 were different names used to denote *LILR* genes in humans (65, 66). In 2004, however, the *LILR* gene nomenclature was officially standardized and accepted by the HUGO Gene Nomenclature Committee (HGNC) (33, 67). LILR family members are categorized as activating (termed LILRA1 to LILRA6) or inhibitory receptors (termed LILRB1 to LILRB5). For *LILR* in non-human primates (NHP), the human *LILR* gene nomenclature is loosely followed by annotation algorithms such as those from NCBI and ENSEMBL. Due to the high levels of similarity between the different *LILR* genes, difficulties may arise in phasing the genomic regions, and therefore it may accidently happen that orthologous genes are not given the corresponding name. To root out such errors, we have selected the latest and freely accessible genomes, which were sequenced by the latest next generation platforms (**Table S2**). The *LILR* region including *RPS9* and *KIR* was extracted from NCBI genome data viewer, and, if available, ENSEMBL database. In **Table S3**, the genes located within the *LILR* region are listed, and the coordinates of these genes on the reference genome are indicated with specific LOC numbers. Available genomic DNA and mRNA sequences were downloaded from the NCBI database and compared to the human *LILR* sequences using Geneious Prime 2020.2.4. Non-human primate genomic DNA sequences were aligned using the MUSCLE method (standard settings, eight iterations) against human genomic sequences. However, different intron length resulted in alignment issues. Therefore, all available primate mRNA sequences were aligned, and a phylogenetic tree analysis was performed using the Geneious tree builder (Jukes-Cantor, Neigbor-Joining, no outgroup, resample tree, bootstrap, random seed = 724,574, number of replicates = 100, create consensus tree, support threshold % = 50). Each cluster with mRNA sequences was aligned and compared to identify transcripts with a potentially incorrect gene name. These sequences were then aligned against the relevant human reference transcripts to sort out whether the genes were annotated correctly or incorrectly in the public database. Next the genomic gene location was defined in relation to the human *LILR* region, before adjusting the gene nomenclature in the relevant non-human primate species. Using this approach, we have explored all available *LILR* data on different nonhuman primate species: namely, chimpanzee, bonobo, gorilla, orangutan, gibbon, rhesus and cynomolgus macaque, and common marmoset, and have introduced a naming of *LILR* genes based on the orthologous positions in the human genome, which was taken as a reference (**Tables S2**, **S3**). Moreover, during this study we have reannotated several of the LOC ID’s that originally comprised two gene entities (**Table S3**). For the chimpanzee and bonobo, we have renamed an additional *LILRA2*-like gene probably encoding for a bona fide gene product (LOC 100612450 and LOC117974252, respectively). This gene locates in the centromeric region between *LILRB2* and *LILRA3*, and shares 99.3% similarity between the two species. Comparative sequence analysis revealed that the domain and intermediate intron sequences of this additional *LILRA2*-like gene are approximately 94% similar to the adjacent *LILRB2* gene. The sequence encoding the domains is comparable with *LILRB2*, while the stem and transmembrane region are more similar to activating LILR receptors; therefore, we have designated this gene as *LILRAB2*. Although, the stem and transmembrane region is comparable to activating LILR receptors, we were not able to pinpoint which recombination event(s) generated the *LILRAB2* gene. There is no evidence for the presence of an additional, maybe disrupted, human *LILRA2* gene in the centromeric region. The reannotation of LOC109024105 for gorilla, LOC100432416 for orangutan, and LOC718403 for rhesus macaque revealed evidence for the presence of a *LILRAB2* gene in these species (**Table S3**). For gorilla, however, the transcription status for this gene is questionable because the current genomic sequence shows a mutation in the transcription initiation site. At this stage we are not certain about the presence or absence of the *LILRB2* and *LILRAB2* genes in the cynomolgus macaque genome. Additionally, in the non-human primates that we have analyzed *LILR* genes that might encode for activating receptors are located at the same position as human pseudogenes *LILRP1* and *LILRP2*. These activating genes belong to a divergent lineage of the ancestral *LILRA1* gene (**Figure 3C**), and we have denoted them as *LILRA7* and *LILRA8* in non-human primates (2).

The *KIR* nomenclature has already been standardized in humans and NHP (68–70). In humans and great and lesser apes, the first *KIR* gene located at the boundary of the *KIR* and *LILR* region is *KIR3DL3*. In rhesus and cynomolgus macaques, *KIR3DL20*, which was previously designated as *KIR3DL2*, is located at the corresponding position and depicted in relevant schemes (12, 71). Furthermore, the *LAIR* gene family consists of two genes that are easily differentiated, and therefore no nomenclature conflicts are reported.

## Comparative Genomics of the *LILR* Region in Great and Lesser Apes

**Figure 4:**
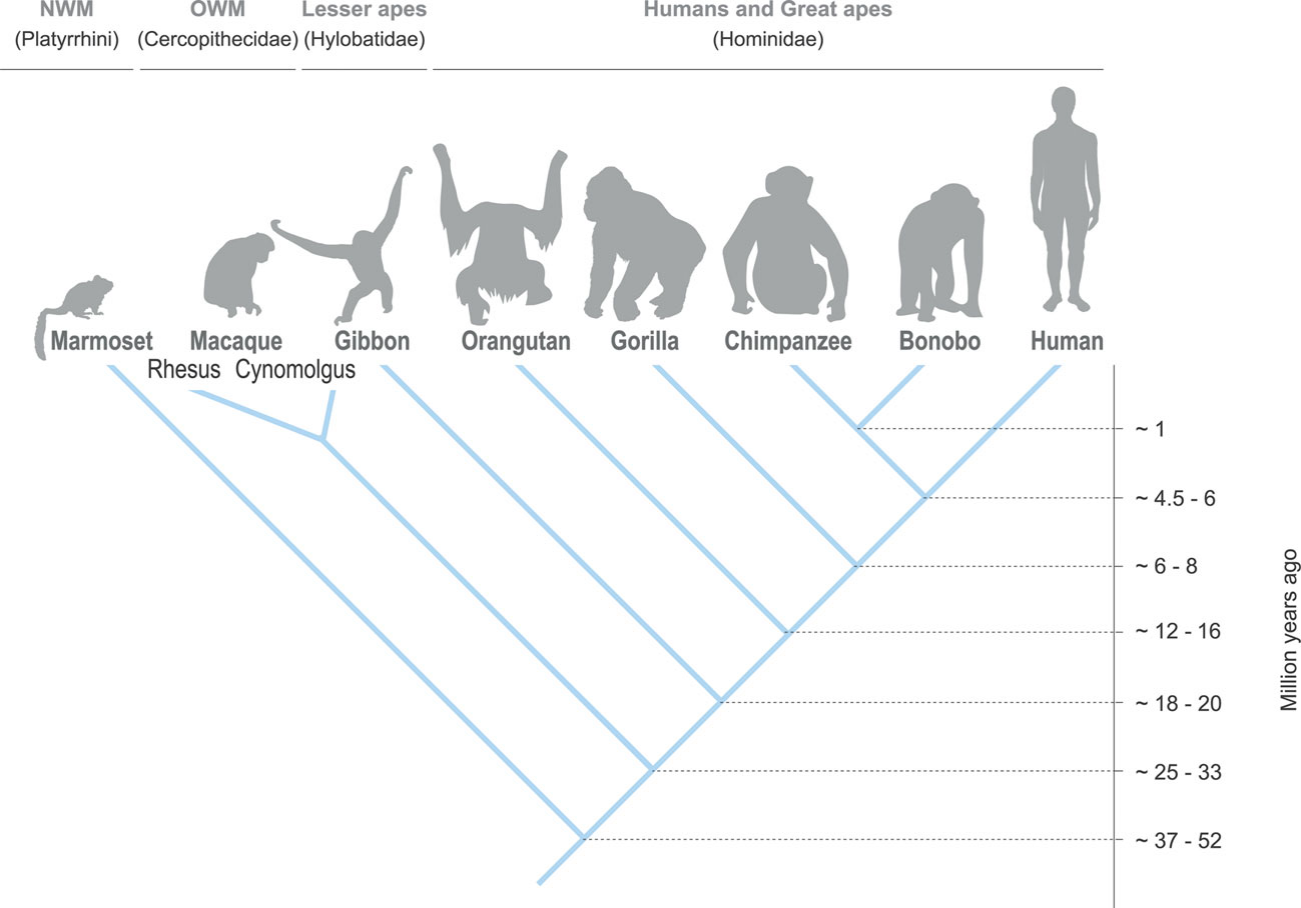
Evolutionary relationship between the different primate species.

The chimpanzee and bonobo, gorilla, and orangutan are all great ape species, and they shared a common ancestor with the human lineage approximately 4.5-6, 6-8, and 12-16 mya, respectively (**Figure 4**) (72–75). For the purpose of this communication, the reference genomes of the chimpanzee, bonobo, western lowland gorilla, and Sumatran orangutan were extracted from the NCBI database and their *LILR* gene organization was thoroughly compared and analyzed using both genomic and mRNA sequences with the human *LILR* region as reference (**Tables S2**, **S3**) (76, 77).

**Figure 5:**
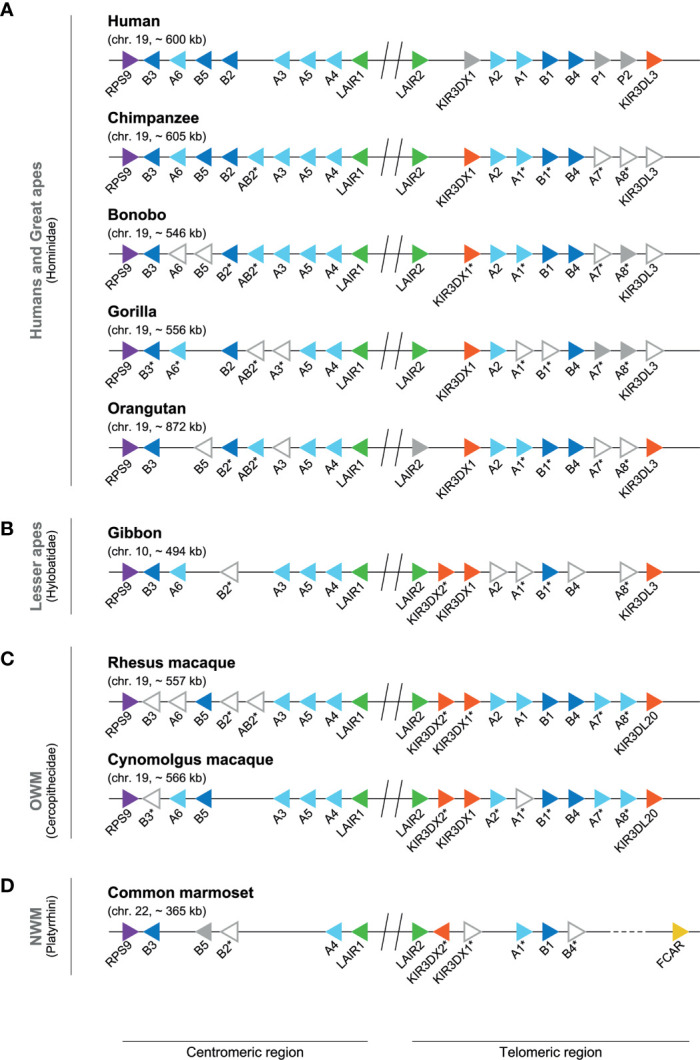
Genomic organization of the *LILR* region in different primate species. Genomic organization of the *LILR* region in human and great apes **(A)**, lesser ape **(B)**, and representatives of the OWM **(C)** and NWM **(D)** species (**Table S2**). Each different *LILR* gene present on a genome is indicated by a colored arrow aligned in such a way that it points in the direction of transcription: *RPS9* (purple), *LILRA* (light blue), *LILRB* (dark blue), *LAIR* (green), *KIR* (red), *FCAR* (yellow), and pseudogenes or genes indicated by NCBI as non-functional, such as bonobo *LILRA8* (grey). The arrows with only a gray outside line represent genes that are indicated by NCBI with low quality protein (**Table S3**), and the expression status of these genes is currently uncertain. The sequence of cynomolgus macaque *LILRA1*, found after reannotation of *LILRA2* (**Table S3**), shows only a correct alignment up to the exon encoding for the third domain structure, and most likely represents a pseudogene. The absence of *LILRB2* and *LILRAB2* on the reference genome of cynomolgus macaques might be an assembly error, as a stretch of approximately 35 kb is identified in this region, which contains an unrelated gene (**Table S3**). The central region is indicated by an interception with two vertical lines. The dotted line (common marmoset) illustrates a large genomic region between *LILRB4* and the *FCAR* lacking annotation. The genes indicated with an asterisk have been renamed using standardized *LILR* gene nomenclature (previously used names are listed in **Table S3**), and particular of these genes were found by re-annotation (**Table S3**).

In great apes, the *LILR* gene region is fixed on chromosome 19, and includes the flanking genes *RPS9* and *KIR3DL3*. In general, the human (~ 600 kb) and chimpanzee *LILR* region (~ 605 kb) are highly similar (**Figure 5**), and orthologs have been reported; they include *LILRA2*, *LILRA4*, *LILRB4*, and *LILRB5* (78, 79). In contrast to humans, however, the chimpanzee *LILR* region contains a *LILRAB2* gene in the centromeric region. At the same position as the human pseudogenes *LILRP1* and -*P2*, two genes are identified in chimpanzees, named *LILRA7* and *LILRA8* (**Figure 5**). These sequences are, however, defined as low quality and modeled from the genome sequence (**Table S3**), and thus might feature inactive genes, comparable to the human pseudogene tandem. In the bonobo, the *LILR* organization (~ 546 kb) is similar to that of the chimpanzee, but the transcription status of several genes, including *LILRA6*, *LILRB5* and *LILRA7*, could not be confirmed. The *LILRA8* gene appears to be non-functional, as sequence analysis revealed two frameshift mutations that result in the introduction of a premature stop codon, and, therefore, this gene is considered to be a pseudogene (**Figure 5** and **Table S3**). The centromeric *LILR* region in gorillas seems to lack *LILRB5*, while the transcription status for four other *LILR* genes is uncertain. The start codon of *LILRA1*, for instance, seems to be interrupted by an insertion. Inactive orthologs of the *LILRA7* and *LILRA8* genes are identified in the telomeric region. The inactivation of *LILRA7* is a result of a mutation in the exon encoding the first Ig-like domain, resulting in a premature stop codon, while *LILRA8* became non-functional due to a similar inactivation event as described in human *LILRP2*. The orangutan centromeric region lacks a *LILRA6* gene, while *LILRB5, LILRA3, LILRA7* and *LILRA8* are defined as low quality sequences (**Figure 5** and **Table S3**). In the telomeric region near the haplotype center, three additional *KIR* genes are annotated in the orangutan reference genome, and a *FCAR* gene could be identified adjacent to them. This region (~ 240 kb) most likely represents an assembly error introduced by the shotgun approach that is used to sequence the reference genome and is therefore not illustrated in **Figure 5**.

The hylobatidae – also known as the lesser apes, and to which the Northern white-cheeked gibbon belongs – shared a common ancestor with humans approximately 18-20 mya (**Figure 4**) (75). The gibbon *LILR* region is located on chromosome 10, and comprises approximately 494 kb. As compared to other primate species, gibbons frequently feature chromosome re-arrangements (80, 81). In the gibbon, part of an ancestral chromosome 19 was re-arranged and incorporated in a reversed form into chromosome 10 (81), which probably resulted in shifting the *LILR* region toward chromosome 10. In comparison to humans, the centromeric region in the gibbon lacks *LILRB5*, while *LILRA7* is absent in the telomeric region (**Figure 5**). For five *LILR* genes, the transcription status is uncertain. Moreover, a second *KIR3DX* gene is observed within the telomeric region, and has been denoted as *KIR3DX2*.

## Genomic Organization of the *LILR* Region in Old and New World Monkeys

The cercopithecidae, also known as Old World monkeys (OWM), shared a common ancestor with humans approximately 25-33 mya (**Figure 4**) (75, 82). The majority of the OWM species can be found in Africa. Most macaque species, however, inhabit various parts of Asia, and one species, the Barbary macaque, lives on the island of Gibraltar in Europe as well as in sections of Northern Africa. Genomes of the Indian-origin rhesus macaque and cynomolgus macaque originating from Tinjil island were available at the NCBI Genome data viewer and Ensembl release 103 and 102, respectively, and were manually explored and annotated for the *LILR* region make-up (**Tables S2**, **S3**) (76, 77). Both the NCBI and Ensembl developed their own unique automatic annotation pipeline, which may result in minor differences between the assemblies. The *LILR* region of rhesus and cynomolgus macaques is located on chromosome 19, and spans approximately 557 and 566 kb, respectively. In rhesus macaques and humans, the make-up of the centromeric *LILR* region appears to be highly similar. The rhesus macaque genome seems to contain, however, a *LILRAB2* gene that might be an orthologue of the chimpanzee and bonobo *LILRAB2*, while it is not certain whether this gene, as well as *LILRB2*, is present or absent in cynomolgus macaques (**Figure 5**). The *LILRA7* and *LILRA8* genes, located at the same position as the equivalent pseudogenes in humans, seem to encode bona-fide activating receptors in macaques, as in-frame transcripts are expected to be transcribed from these genes. In rhesus macaques the transcription status of *LILRB3*, *LILRA6*, and *LILRB2* is uncertain. In cynomolgus macaques, the *LILRA1* gene may be disrupted by an insertion subsequent to exon 5, which indicates a secreted gene product or an inactive copy, and the transcription status of *LILRB3* is uncertain. In addition, like found in the gibbon, also the telomeric region of macaques contains an additional gene that belongs to the *KIRDX* lineage, termed *KIR3DX2*.

Platyrrhini, or New World monkeys (NWM), shared with humans a common ancestor that lived approximately 27-52 mya (**Figure 4**) (75, 82). The common marmoset is likely the most prominently studied NWM organism; its genome is available at the NCBI Genome data viewer, and it was manually explored and annotated for the *LILR* region (**Table S2**, **S3**) (76, 77). The common marmoset *LILR* region is located on chromosome 22, and spans approximately 365 kb. Comparative karyotyping of the chromosomes of common marmosets and humans showed the homology of marmoset chromosome 22 with human chromosome 19 (83). In marmosets, both *LILR* regions, centromere and telomere, show a substantial contraction with regard to the number of genes as compared to the human *LILR* region (**Figure 5**). The centromeric region contains *LILRB3*, *LILRB5* (which became a pseudogene), *LILRB2*, and *LILRA4*. In the telomeric region *LILRA1*, *LILRB1*, and *LILRB4* were encountered. *LILRB2* and *LILRB4* are, however, defined as low quality sequence. Furthermore, a tandem of *KIR3DX1* and *KIR3DX2* genes was observed. Adjacent to *LILRB4*, an unannotated stretch of ~ 280 kb is identified, which does not contain a *LILR* or *KIR* equivalent. This might reflect an error in the assembly of the reference genome.

To the best of our knowledge, we present here the first comprehensive comparison of the *LILR* region in different primate species. We should note, however, that for most non-human primate species only one or a few complete genomes have been sequenced. The *LILR* organization in this study is based on a single reference genome per species. Although the *LILR* cluster seems to be an ancient and conserved set of genes, subtle variation in the gene organization might exist per individual or per population, as is demonstrated for the null haplotype in humans that lacks the presence of *LILRA3*. Moreover, the transcription status of several genes was also not confirmed on the current reference genomes and requires additional characterization studies before definitive conclusions can be drawn.

## A *KIR3DX* Gene Tandem in Lesser Apes and Old and New World Monkeys

In primates and cattle, two distinct *KIR* gene clades, *KIR3DL* and *KIR3DX*, have been defined (55, 84). The *KIR3DL* lineage is duplicated, and generated a *KIR* gene family in simian primates, while the *KIR3DX* lineage in cattle was subjected to expansion, resulting in a functional *KIR* gene family (84, 85). It is hypothesized that an ancestral *KIR* gene emerged approximately 135 mya before radiation of the placental mammal resulting in *KIR3DL* and *KIR3DX* daughter genes. In primates, the *KIR3DX* gene is embedded within the *LILR* region, while the *KIR* gene family is located telomeric of the *LILR* gene family (**Figure 1**). In humans, *KIR3DX1* is regarded as a pseudogene due to a 7 bp deletion at the end of exon 5, resulting in the introduction of a premature stop codon in exon 7, and the frameshift was confirmed in 86 healthy individuals (55). Although human *KIR3DX1* is classified as a pseudogene by HUGO, *KIR3DX1* cDNA could be cloned from a human NK cell line (NK-92), suggesting that transcription of the gene may occur (55).

**Figure 6:**
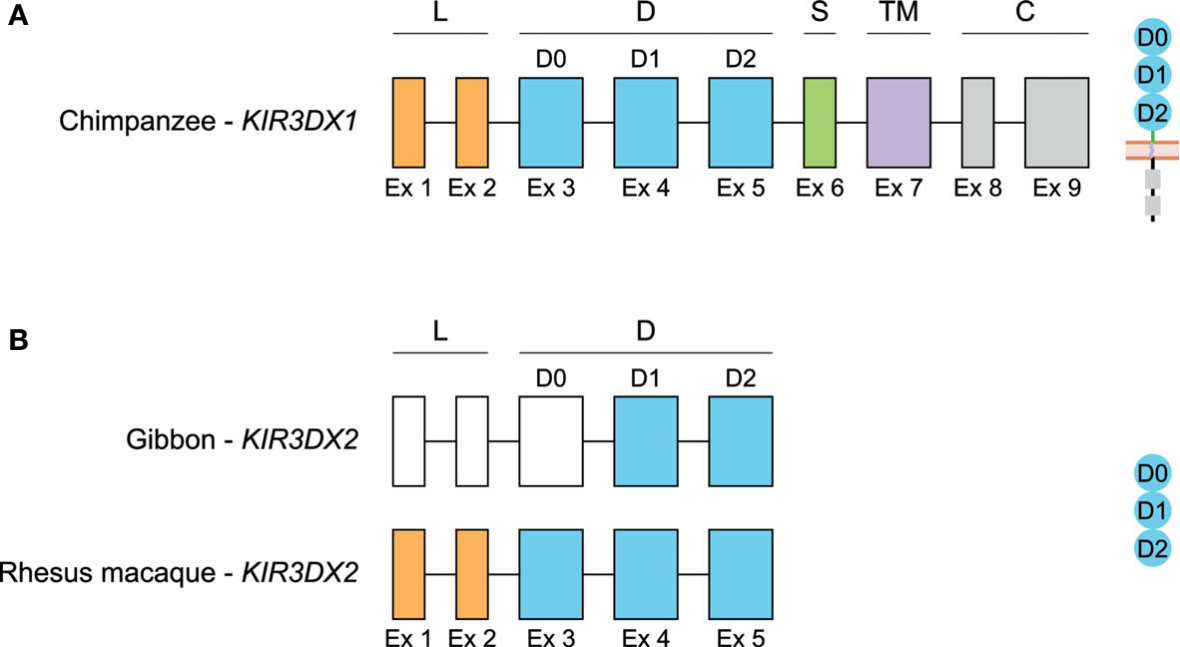
Genomic organization of *KIR3DX1* and *KIR3DX2* genes in primates. Schematic illustration of the genomic organization of the chimpanzee *KIR3DX1* gene that is representative for human, bonobo, gorilla, orangutan, gibbon, rhesus and cynomolgus macaque, and common marmoset **(A)**, and of the *KIR3DX2* gene in gibbon and rhesus macaque, of which the latter is representative for cynomolgus macaque and common marmoset **(B)**. Exons 1 and 2, encoding the leader peptide (L), are depicted in orange; exons 3-5, encoding Ig-like domains (D), are in blue; exon 6, encoding the stem (S), is in green; exon 7, encoding the transmembrane (TM) region, is in purple; and exons 8 and 9, encoding the cytoplasmic tail (C), are in grey. Even though the start codon of gibbon *KIR3DX2* is identified, the first three exons could not be defined, and are therefore represented by white boxes. The putative corresponding protein structures are schematically depicted adjacent to the genomic organization.

In the genomes of several NHP species analyzed, we observed a second *KIR3DX* gene that most likely arose by an ancient duplication event of *KIR3DX1*, and the sister gene has been termed *KIR3DX2* (**Figures 5**, **6**). The NCBI database classifies the *KIR3DX2* as a protein coding gene, but it lacks the exons encoding the transmembrane and signaling regions, and therefore may encode a secreted gene product (**Figure 6B**). In marmoset, the two gene copies are arranged head-to-head (**Figure 5**), and it is postulated that this orientation arose due to a species-specific duplication event (55). However, we observed two *KIR3DX* copies arranged head-to-tail in the lesser apes and OWMs, suggesting that both gene entities might already be present in a common ancestor with NWM and remained conserved, while one of the *KIR3DX* genes was lost during evolution of the human and great ape lineage. From this perspective, the head-to-head arrangement in common marmoset might be a genome assembly failure, and the orientation should be head-to-tail as well. Additional genome analysis must be performed to sort out if this arrangement is a species-specific duplication event or an assembly failure. The presence of two related *KIR3DX* genes is, however, not specific for macaques residing in Asia, but can be found in the NCBI database available genomes of olive baboon and green monkey as well (data not shown), both of which inhabit Africa.

*KIR3DX1* exon sequences are compared with chimpanzee *KIR3DX1* using Geneious Prime 2020.2.4, since the full-length human *KIR3DX1* transcripts would contain the 7 bp deletion, and the chimpanzee is most closely related to humans (**Table S4**) (86). Comparative analysis of the exons revealed a high level of interspecies sequence similarity to chimpanzee *KIR3DX1* for gorilla (97.9%), orangutan (95.1%), gibbon (94.3%), rhesus macaque (90.8%), and cynomolgus macaque (91.0%). In contrast, *KIR3DX1* in common marmoset is 68.4% similar to chimpanzee *KIR3DX1*. Furthermore, the coding region of *KIR3DX1* in rhesus and cynomolgus macaque is 99.4% similar, indicating that this gene is highly conservated in macaque species. When introns are included in the sequence similarity comparison, higher levels of diversity are determined. In orangutans, for instance, the genomic *KIR3DX1* sequence displays 83.5% similarity to chimpanzees, and the difference are mainly explained by insertions and deletions in the introns. The genomic sequence of chimpanzee *KIR3DX1* is 76.6% and 78.7% similar to the sequences of rhesus and cynomolgus macaques, respectively. These observations suggest a selective pressure to largely conserve the *KIR3DX1* exons, whereas the introns might be more prone to diversification, which may impact the regulation of expression levels and transcript splicing. Overall, the *KIR3DX1* coding sequence is highly conserved between human, great apes, lesser apes, and OWM, whereas the *KIR3DX1* gene in NWM diverged during evolution. The function of KIR3DX1 is still unknown. However, it has an inhibitory potential due to the presence of two ITIM motifs in the cytoplasmic tail (55). Full-length *KIR3DX1* transcripts were identified in peripheral blood mononuclear cells of rhesus macaques, in addition to an isoform that lacked exon 5, but quantification indicated low expression levels (55). To date, no ligand for KIR3DX1 has been reported.

Comparing the sequences of *KIR3DX2*, up to exon 5 that encodes the third extracellular Ig-like domain, more species-specific variation is observed. Taking the rhesus macaque sequence as most well-defined reference, the gibbon *KIR3DX2* only shows 73.1% and 35.9% similarity at the exons and genomic level, respectively. Cynomolgus macaques, which shares a relatively close common ancestor with the rhesus macaques approximately 1-3 mya, have almost an identical *KIR3DX2* gene in the coding (99.9%) and genomic (99.5%) sequence regions. In marmosets, the *KIR3DX2* gene largely deviated at the genomic DNA level, with 57.5% similarity to rhesus macaques, whereas the exon sequences were more conserved (80.4%).

The most likely explanation for an additional *KIR3DX* gene in lesser apes, OWM and NWM is a duplication of *KIR3DX1* in a common ancestor that is lost during radiation towards human and great apes. The *KIR3DX2* sequence in gibbons is, however, far from similar to *KIR3DX1* in chimpanzees (59.4% on exons and 40.6% on gDNA). In contrast, the coding regions of *KIR3DX2* in both macaque species and in the common marmoset seem to be more conserved, with 83.7/83.8% and 85.7% sequence similarity to the coding sequence of *KIR3DX1* in chimpanzees, respectively. This substantiates an ancient *KIR3DX1* duplication event, which remained more conserved in OWM, while the duplicated gene diverged during evolution in gibbons.

## LILR Gene Function in Humans

In humans, LILR receptors are expressed both on myeloid and lymphoid immune cells, including monocytes, B and T lymphocytes, natural killer (NK) cells, neutrophils, and dendritic cells (DC), and it is generally accepted that they control a variety of immune responses and maintain homeostasis (54, 59, 87, 88). Each LILR receptor is expressed on a unique repertoire of cell populations (**Table 1**). Inhibitory LILR receptors probably function as immune checkpoints by screening and eliminating manipulated immune cells, which lack HLA class I expression, as depicted in the “missing self” hypothesis (19, 59, 107). For example, LILRB1 interaction with MHC class I molecules may regulate cell phenotype and function (108). Several immune checkpoint receptors are present in the human immune system, including programmed cell death protein-1 (PD-1). PD-1 is not a member of the IgSF superfamily, but the intracellular signal transduction is equal to inhibitory LILR receptors, resulting in negative regulation of the immune system. Furthermore, the inhibitory receptor LILRB2 regulates neuronal functions such as axonal regeneration and synaptic plasticity (57–59). Interaction between LILRB2 and ANGPTL molecules might play a role in angiogenesis, although its precise role is at present poorly understood (56). Both LILRB1 and LILRB2 interact with HLA-G, a non-classical MHC class I molecule, resulting in a dominant immunosuppressive effect that plays a role in pregnancy as well as in transplant tolerance, infection, and cancer (6, 109–111).

**Table 1:** Cell distribution and ligands of human LILR receptors.

Receptor	Type	Cell distribution	Ligand	Reference
LILRA1	Class I	Monocytes, B-cells, mast cell progenitor	Classical HLA class I	(89, 90)
LILRA2	Class I	Monocytes, macrophages, T-cells, NK cells, DC, eosinophils, basophils, neutrophils, granulocytes, mast cell progenitor	Microbially cleaved antibodies, soluble HLA class I molecules	(90–94)
LILRA3	Class I	Monocytes, B-cells, T-cells, NK cells, neutrophils	HLA-C, HLA-G	(62, 90, 94, 95)
LILRA4	Class II	DC	BST2	(96)
LILRA5	Class II	Monocytes, neutrophils	Unknown	(94, 97)
LILRA6	Class II	Monocytes	Unknown	(98)
LILRB1	Class I	Monocytes, B-cells, T-cells, NK cells, DC, eosinophils, neutrophils, mast cell progenitor	HLA class I (classical and non-classical), UL18, S100A9, *Staphylococcus aureus*, *Escherichia coli*, RIFIN	(65, 66, 89, 90, 94, 96, 99–101)
LILRB2	Class I	Monocytes, macrophages, DC, eosinophils, neutrophils, mast cell progenitor	HLA class I (classical and non-classical), ANGPTL, CD1, nogo66, beta-amyloid	(89, 94, 102, 103)
LILRB3	Class II	Monocytes, macrophages, DC, eosinophils, basophils, neutrophils, granulocytes, mast cell progenitor	Unknown	(89, 90, 93, 94, 98)
LILRB4	Class II	Monocytes, macrophages, DC, mast cell progenitor, plasmablast	ALCAM/CD166	(89, 90, 96, 101, 103–105)
LILRB5	Class II	Monocytes, T-cells, NK cells, mast cells	HLA-B27 free heavy chain, ANGPTL	(90, 106)

Cellular distribution on immune cells and corresponding ligands according to the literature. DC, dendritic cells; NK, natural killer cells; HLA, human leukocyte antigen; BST2, bone marrow stromal cell antigen 2; RIFIN, repetitive interspersed family; ANGPTL, angiopoietin-like protein; and ALCAM, activated leukocyte cell adhesion molecule.

**Figure 7:**
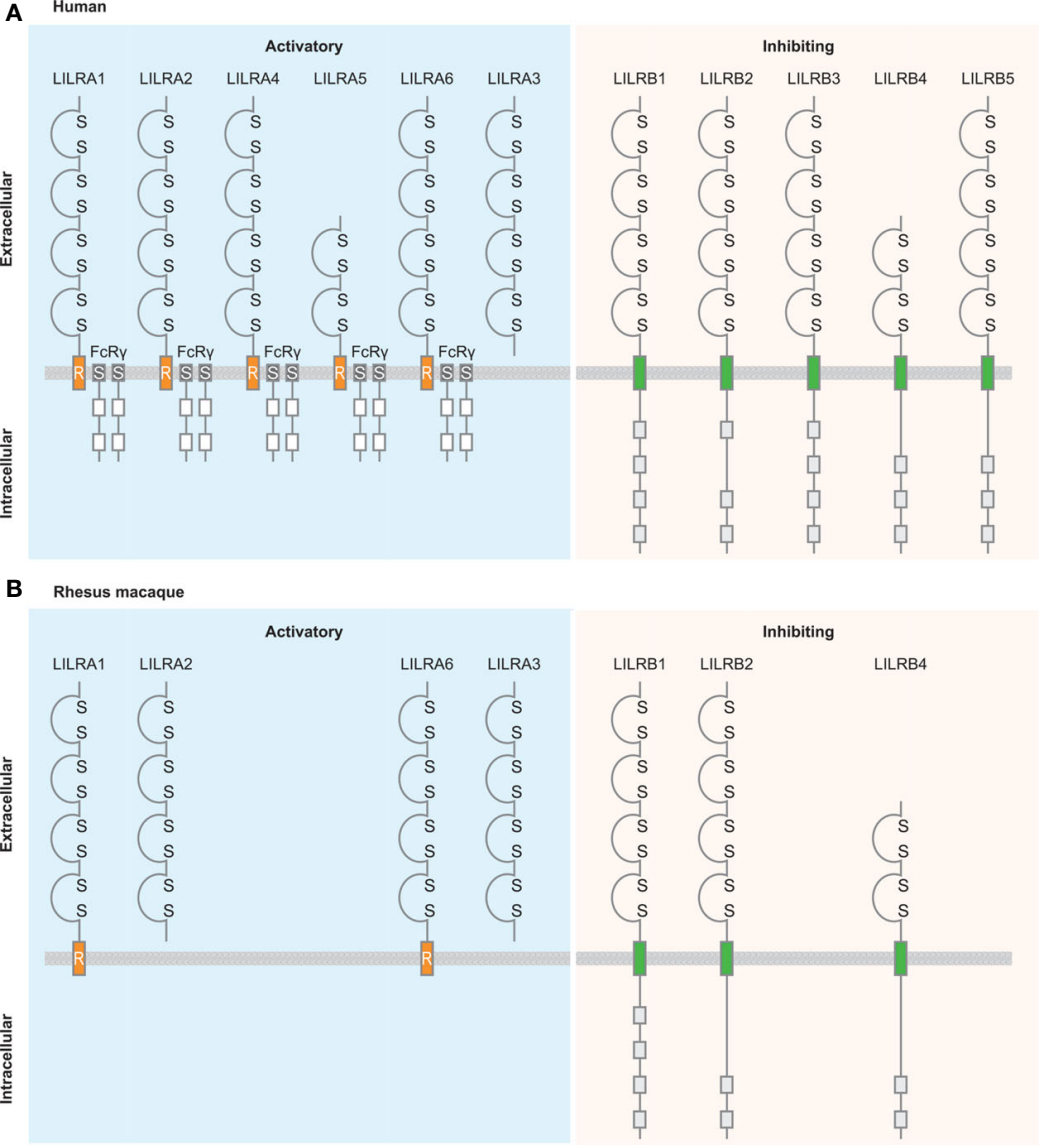
Schematic illustration of the structure of LILR family members in human and rhesus macaque. A structural comparison between LILR family members in humans **(A)** and rhesus macaques **(B)** (112). LILR receptors are classified based on the presence or absence of the transmembrane region and the cytoplasmic tail. Activating receptors associate with the γ-chain of Fc receptors, while the inhibitory receptors contain cytoplasmic immunoreceptor tyrosine-based inhibition motifs. Based on the conserved nature of the LILRA receptors it is plausible that also in rhesus macaque these types of receptors associate with FcRγ, however, because this is not officially documented it is not illustrated in the figure. LILRA3 is a soluble receptor lacking the transmembrane region and cytoplasmic tail. Ig-like domains are indicated by a domain structure containing disulfide bridges; transmembrane region is indicated by orange boxes including an R, which refers to the presence of the positively charged arginine residue, for the activating LILR receptors and by green boxes for the inhibitory LILR receptors; Fc receptor transmembrane by dark grey boxes containing an S; immunoreceptor tyrosine-based activation motifs by white boxes; and immunoreceptor tyrosine-based inhibition motifs by light-grey boxes.

Activating and inhibitory LILR receptors consist of two or four extracellular Ig-like domains (**Figure 7A**) (33). In addition, LILR receptors are classified based on amino acid sequence similarity in the ligand binding sites, distinguishing class I and II type receptors (**Table 1**). Class I includes LILRA1, LILRA2, LILRA3, LILRB1, and LILRB2, and interacts with classical and non-classical HLA class I molecules (88). Class II includes LILRA4, LILRA5, LILRA6, LILRB3, LILRB4, and LILRB5, and seems to have ligands other than HLA molecules. An exception is formed by LILRB5, which interacts with ANGPTL, but binding to HLA class I heavy chains was recently reported as well (88, 113). As is known so far, class II receptors appear to interact with one or two specific ligands, like bone marrow stromal antigen 2 (BST2/CD317) and an activated leukocyte cell adhesion molecule (ALCAM/CD166), while class I receptors can interact with a broader repertoire of HLA-ligands (35, 88, 114–116). Caution should be exercised in this regard, however, because the ligands of LILRA5, LILRA6, and LILRB3 are unknown at present.

Activating LILR receptors do not possess a cytoplasmic tail that has the capacity to transduce a signal and therefore associate with the γ-chain of Fc receptors *via* a positively charged arginine residue in the transmembrane region of the LILRA receptor (114, 117). Immunoreceptor tyrosine-based activation motifs (ITAM) located in the γ-chain of Fc receptors are phosphorylated, and activate downstream activation pathways (118). Inhibitory LILR receptors have a cytoplasmic tail containing three or four immunoreceptor tyrosine-based inhibitory motifs (ITIM), which downregulate cell activation by recruiting phosphatases of the Src kinase family (119–121). Phosphorylated ITIM motifs serve as docking sites for other enzymes such as Src homology region 2 domain-containing phosphatase-1 (SHP-1), Src homology region 2 domain-containing phosphatase-2 (SHP-2), and Src homology 2 domain containing inositol polyphosphate 5-phosphatase 1 (SHIP), resulting in the negative regulation of immune cell activation. The fine balance between activating and inhibitory LILR receptors is necessary for the modulation of immune responses and the maintenance of immune cell homeostasis.

## LILR Receptors in Non-Human Primates

To date, nine chimpanzee *LILR* genes have been thoroughly characterized, of which eight seem to encode for a functional protein, seven with four Ig-like domains and one with two Ig-like domains (78). As expected, the position of the cysteine residue essential for disulfide bridge formation in the domains is conserved between humans and chimpanzees. Comparable with humans, the activating LILR receptors in chimpanzees contain an arginine residue in the transmembrane region, while the inhibitory LILR receptors in chimpanzees have a cytoplasmic tail containing three or four ITIM motifs.

In rhesus macaques, LILR receptors comprise the same characteristics as human LILR receptors, including two or four Ig-like domains, the presence of a positively charged arginine residue in the transmembrane region of activating LILR, and the intracellular region of inhibitory LILR containing two or four ITIM motifs (**Figure 7B**) (112).

As far as we know, ligand-binding studies have not been conducted for chimpanzee and rhesus macaques. LILR/MHC dynamics was, however, studied recently in HIV infection in humans and compared to an early SIV-infection model in cynomolgus macaques (122). A monoclonal antibody specific for the extracellular part of human LILRB2 showed cross-reactivity with cynomolgus macaque cells. In humans and cynomolgus macaques, the expression of LILRB2 and a LILRB2-like protein, respectively, was shown on a similar immune cell subset, which included monocytes, classical DC, plasmacytoid DC, and polymorphonuclear leucocytes. Overall, the data illustrated that during a SIV/HIV-infection the cynomolgus macaques LILRB2-like protein seems to negatively regulate the same immune cell population as human LILRB2. So far, MHC class I is the only known ligand engaging with cynomolgus macaque LILRB2.

Considering the conserved LILR function in humans, and the structural similarities reported for chimpanzees and rhesus macaques, it is tempting to speculate that NHP LILR receptors engage with MHC equivalents of HLA ligands (45, 78, 112, 122).

## Role of Human LILR Receptors in Health and Disease

In healthy individuals and during pregnancy, the modulation of immune activity is tightly regulated by a complex mechanism that involves different members of the *HLA* family and multiple regulatory gene systems, including *LILR, KIR*, and *NKG2*. Pregnancy is an exquisite example of a balanced immune regulation and adaption to protect the embryo against an unintended maternal immune response. Expression of HLA-A and -B is limited during pregnancy to avoid alloreactivity by B and T cells, whereas classical HLA-C and non-classical HLA-E and -G are expressed on fetal trophoblasts (123, 124). During the early stages of pregnancy, a distinctive subset of uterine NK (uNK) cells is involved in placental formation. Different combinations of KIR and HLA-C allotypes regulate these uNK cells, in which the extensive genetic variation of both gene systems associates with successful pregnancy or with complications, such as recurrent miscarriage (125). The role of the *KIR* gene family is reflected by interactions of KIR2DL4 with soluble HLA-G, which has been described (126, 127). In short, these interactions probably modulate the production of cytokines and chemokines to promote vascular remodeling in early pregnancy (128). Activating receptors NKG2D, as well as DNAM-1- and NKp44 mediate the regulation of NK cell and modulate NK cell activity (126, 129). HLA-G expression is restricted to trophoblast cells and might also be recognized by LILRB1, which is highly expressed on NK cells found in the maternal decidua, and by LILRB2, which is expressed on maternal decidual macrophages (124, 130, 131). These mechanisms protect the embryo against NK- directed cell lysis (131). Furthermore, multiple LILR receptors are described as beneficial, resulting in individuals with a protective phenotype against multiple sclerosis, or in individuals who can control virus infections such as HIV-1 (132, 133). However, LILR receptors might play a negative role as well, and are associated with the outcome of several diseases. In some diseases, LILR receptors can be regarded as a genetic risk factor (**Table 2**). Therefore, LILR receptors might be useful as diagnostic markers and a target for immunotherapies (58, 59). The role of LILRs in different kinds of diseases is briefly discussed in the following paragraphs. We would like to emphasize, however, that these mainly concern diseases with an immunological component that we have highlighted here exclusively in the context of LILR.

**Table 2:** Overview of human LILR receptors and their associations with disease.

Receptor	Type	Disease	Reference
LILRA1	No disease associations documented according to literature		
LILRA2	Autoimmune and autoinflammatory diseases	Rheumatoid arthritis	(134)
		Systemic lupus erythematosus	(135)
		Microscopic polyangiitis	(135)
	Infectious diseases	Hansen’s disease	(136)
LILRA3	Autoimmune and autoinflammatory diseases	Rheumatoid arthritis	(95, 137, 138)
		Systemic lupus erythematosus	(137)
		Sjögren’s syndrome	(139)
		Ankylosing spondylitis	(140)
		Intestinal bowel disease	(141)
	Neurodegenerative disorders	Multiple sclerosis	(133, 142, 143)
	Cancer	Prostate cancer	(144)
LILRA4	No disease associations documented according to literature		
LILRA5	Autoimmune and autoinflammatory diseases	Rheumatoid arthritis	(134, 145)
LILRA6	Autoimmune and autoinflammatory diseases	Atopic disease	(146)
LILRB1	Autoimmune and autoinflammatory diseases	Rheumatoid arthritis	(147)
		Systemic lupus erythematosus	(148)
		Ankylosing spondylitis	(149)
	Infectious diseases	Pulmonary tuberculosis	(150)
		CMV	(99, 151)
		Dengue	(152, 153)
		Malaria	(154)
		HIV	(132)
	Cancer	Non-small cell lung cancer	(155)
		Leukemia	(156)
		Gastric cancer	(157)
LILRB2	Autoimmune and autoinflammatory diseases	Rheumatoid arthritis	(134)
	Neurodegenerative disorders	Alzheimer’s disease	(57, 58)
	Infectious diseases	*Salmonella* infection	(158)
		HIV	(159)
	Cancer	Colorectal cancer	(160)
LILRB3	Autoimmune and autoinflammatory diseases	Rheumatoid arthritis	(134)
		Takayasu’s disease	(161)
LILRB4	Autoimmune and autoinflammatory diseases	Systemic lupus erythematosus	(162)
	Infectious diseases	*Salmonella* infection	(158)
	Cancer	Gastric cancer	(157)
		Leukemia	(163, 164)
LILRB5	No disease associations documented according to literature		

## Autoimmune and Autoinflammatory Diseases

Rheumatoid arthritis patients abundantly express LILRA2, LILRA3, LILRA5, and LILRB2, with the presence of LILRA2, LILRA5, and LILRB2 significantly correlating with disease activity (95, 134, 145). The underlying mechanisms are not yet fully understood, but it is postulated that disrupted gene expression may contribute to an excessive inflammatory immune response. In addition, insufficient inhibitory signaling as a result of single nucleotide polymorphisms (SNP) in the promotor region or of post-transcriptional regulation might contribute to rheumatoid arthritis susceptibility (147). LILRA3 is identified as a genetic risk factor for rheumatoid arthritis, systemic lupus erythematosus, and Sjogren’s syndrome (137–139). In systemic lupus erythematosus patients, disrupted LILRB1 expression and/or deficient inhibitory signaling is observed (148). This observation is comparable with the postulated cause of the excessive inflammatory immune response in rheumatoid arthritis patients. In addition, polymorphisms may impact disease association. Polymorphisms found in *LILRB4* resulted in a loss of function, and, as a consequence, increased inflammatory cytokine levels in systemic lupus erythematosus were observed (162). *LILRA2* splice site polymorphism affects alternative splicing, resulting in a different isoform, which is associated with systemic lupus erythematosus and microscopic polyangiitis (135).

Likewise, several LILR disease associations are reported in chronic disorders, and the majority of these associations are genetic. In a cohort of family-related atopic disease patients, a single copy of *LILRA6* might be related to the development of the disease (146). *LILRA3* and *LILRB3* are both identified as susceptibility genes in Takayasu’s arteritis (161). Furthermore, LILRA3 is associated with ankylosing spondylitis susceptibility in different cohorts, including Han Chinese subpopulations and a Polish population, underlying the genetic differences between different ethnicities (140, 149). Associations involving disruptive gene expression are seldom reported. LILRA3 expression is increased in intestinal bowel disease patients, probably resulting in suppression of the anti-inflammatory immune response (141).

## Neurodegenerative Disorders

Human LILRB2 and its murine ortholog PirB interact with soluble β-amyloid, leading to enhanced cofilin signaling, which is observed in the brains of humans with Alzheimer’s disease (58, 165). In a transgenic mice Alzheimer’s disease model, memory deficits in adult mice are caused by PirB deficiency, which results in the loss of synaptic plasticity in the juvenile visual cortex. It is postulated that due to the orthologous relationship between LILRB2 and PirB, LILRB2 may contribute to Alzheimer’s disease neuropathology, and might be a suitable therapeutic target. The *LILRA3 null* haplotype might increase the risk of relapsing multiple sclerosis in Spanish patients (133). These findings were confirmed in German and French multiple sclerosis patients of Caucasian descent (142). In contrast, in a Polish cohort, no association for disease susceptibility was found, but it was shown instead that a *LILRA3* deletion is associated with the later onset of multiple sclerosis (143). In multiple sclerosis patients, LILRB2 and HLA-G are co-expressed on central nervous system cells and in areas with microglia activation, while HLA-G expression is barely detectable in healthy controls (166). LILRB2 and HLA-G play a role in immune reactivity in the central nervous system, which might act as an inhibitory feedback mechanism to downregulate the damaging effect of T-cell infiltration in neuroinflammation.

## Infectious Diseases

The immune response to bacteria often results in increased expression of LILR receptors on the cell surface. For example, in lepromatous patients, LILRA2 is upregulated in the lesions, and suppresses the innate host immune response by shifting cytokine production from interleukin-12 (IL-12) toward IL-10 (136). LILRB1 expression is elevated on CD56^dim^CD16^+^ NK cells during active pulmonary tuberculosis (150). It is postulated that CD56^dim^CD16^+^ NK cells correlate with the disease severity of pulmonary tuberculosis, because CD56^dim^LILRB1+ NK cells are not capable of eliminating infected cells. Also, during *Salmonella* infection, LILRB2 and LILRB4 are upregulated, which results in the expansion of tolerogenic antigen-presenting cells owing to an insufficient response to toll-like receptor signaling (158).

Viruses developed other strategies to dysregulate the host immune response by abusing immune receptors. The most studied viral infection with regard to LILR receptors is cytomegalovirus (CMV) infection, which expresses UL18, an MHC class I homolog, on infected cells. The engagement of UL18 and LILRB1 may inhibit the clearance of CMV-infected cells, and, therefore, CMV might escape the innate immune response (99). During the adaptive immune response, CMV-infected cells expressing UL18 are lysed by CD8+ T cells, while CMV-infected cells lacking UL18 are not eliminated, and therefore CMV might escape this host immune response as well (151). Recurrent CMV infection or deficient immune response is frequently observed in transplant patients. Another example was observed during a dengue infection, where LILRB1 was shown to engage with an unknown dengue virus-related ligand, resulting in the obstruction of FcγR activation and allowing host cell entrance and viral replication (152, 153). Recently, an association with LILRB1 and malaria was observed. *Plasmodium falciparum*, the causative agent of malaria, produces repetitive interspersed family (RIFIN) proteins, which are displayed on infected erythrocytes (167). Some RIFINs interact with LILRB1, which could potentially result in tempering the host immune response by suppressing NK cell function response (154).

## Cancer

In chronic lymphocytic leukemia, a significant increase of LILRB1 expression is detected on NK cells, resulting in a lack of elimination of leukemic cells (156). In acute myeloid leukemia, LILRB4 is expressed on monocytic leukemia cells, generating an immunosuppressive microenvironment contributing to the infiltration of other tissues, including the central nervous system (163, 164). Co-expression of LILRB2 and HLA-G is observed in tissues of human primary colorectal cancer, while different expression patterns of LILRB1 and LILRB4 are observed in gastric cancer patients (157, 160). Differential expression may contribute to the proliferation, migration, and invasion of tumor cells. In addition, genetic risk factors have been reported in different types of cancer. A genome-wide association study in Chinese men revealed that *LILRA3* SNP rs103294 and *LILRB1* SNP rs16985478 may be a risk factor for prostate cancer and non-small cell lung cancer, respectively (144, 155).

## Concluding Remarks

In this communication, we provided an overview on the genomic organization of the *LILR* region in primates with which we illustrate that the *LILR* region remained largely conserved throughout primate evolution (**Figure 5**). Minor differences in gene content were observed, but at this stage it is not clear whether allelic variation influences the complexity of the system. Further research is necessary to arrive at a solid conclusion. By comparing channel catfish, chicken, opossum, primates, mice, cattle, goat, and pig, we estimated that the *LILR* gene family likely emerged more than 450 mya, probably in the same time frame as the MHC system. It is thought that the evolution of the MHC system influenced *KIR* gene evolution (and vice versa), but it is not evident whether it influenced *LILR* gene evolution as well (36, 49, 55, 168). Some LILR receptors engage with the highly conserved α3-domain of MHC class I molecules and the β2m subunit, suggesting that the main function of LILR receptors is immune surveillance by scanning immune cells for the presence or absence of MHC class I. More sophisticated systems, like that of the *KIR* genes, appeared later in evolution, and are able to scan for the presence of polymorphic epitopes on MHC class I molecules. In humans, *KIR3DX1* is classified as a pseudogene, while *KIR3DX1* in the non-human primates is seen to code for a functional gene product. A duplication of *KIR3DX1* was observed in the lesser apes, OWMs, and NWMs, suggesting that this duplication might be present in the ancestor and was lost in great apes and humans. As far as we know, the function and ligand of *KIR3DX1* is not yet resolved. At last, LILR receptors are placed in context for the role they may play in health and disease. It is tempting to speculate that old genes are frequently associated with diseases. However, one would expect that serious disease associations linked to old genes have been weeded out during evolution. The other issue is that disease association in a highly conserved region with limited levels are hard to pick up due to linkage phenomena. Since the *LILR* region in primates is remarkably conserved, non-human primates are an excellent tool to thoroughly study the functional aspects of *LILR* genes. This type of undertaking might enhance the available non-human primate disease models in order to improve the health both of humans and animals.

## Author Contributions

LS drafted the manuscript. JB, NG, and RB edited the manuscript. All authors contributed to the article and approved the submitted version.

## Conflict of Interest

The authors declare that the research was conducted in the absence of any commercial or financial relationships that could be construed as a potential conflict of interest.

## Publisher’s Note

All claims expressed in this article are solely those of the authors and do not necessarily represent those of their affiliated organizations, or those of the publisher, the editors and the reviewers. Any product that may be evaluated in this article, or claim that may be made by its manufacturer, is not guaranteed or endorsed by the publisher.
