# Supplementary material for: The Genomic Organization of the LILR Region Remained Largely Conserved Throughout Primate Evolution: Implications for Health And Disease
Source: Front Immunol. 2021 Oct 19;12:716289. doi: 10.3389/fimmu.2021.716289 (PMC8562567; doi:10.3389/fimmu.2021.716289)
Supplement: Supplementary file 2 [file Table_1.docx]

**Table S1: Overview of the LILR-like receptors in humans and vertebrates other than non-human primates.**

Overview of LILR-like receptors in vertebrates, including nomenclature for activating and inhibitory LILR-like receptors, cell distribution, and ligands. LILR = Leukocyte immunoglobulin-like receptor; PIR = paired immunoglobulin-like receptor; CHIR = chicken immunoglobulin-like receptor; LITR = leukocyte immune-type receptor; PBMC = peripheral blood mononuclear cell; HLA = human leukocyte antigen; and MHC = major histocompatibility complex.

| Species | Activating receptors | Inhibitory receptors | Cellular distribution | Ligand | Reference |
| --- | --- | --- | --- | --- | --- |
| Human | LILRA | LILRB | Myeloid and lymphoid cell lineages | HLA class I | (33) |
| Goat | LILRA | LILRB | Alveolar macrophages, bone marrow macrophages, CD4^+^ T-cells, CD8^+^ T-cells | Unknown | (42) |
| Cattle | LILRA | LILRB | PBMC | Unknown | (43) |
| Mouse | PIRA | PIRB | Macrophages, neutrophils, dendritic cells, mast cells, B-cells | MHC class I | (39,45) |
| Pig | LILRA | LILRB | PBMC | Unknown | (41) |
| Chicken | CHIRA | CHIRB | Myeloid and lymphoid cell lineages | MHC class I | (40,47) |
| Channel catfish | LITR2 | LITR1 | PBMC, cytotoxic T-cells | Putative MHC class I | (46) |
