# Supplementary material for: The Genomic Organization of the LILR Region Remained Largely Conserved Throughout Primate Evolution: Implications for Health And Disease
Source: Front Immunol. 2021 Oct 19;12:716289. doi: 10.3389/fimmu.2021.716289 (PMC8562567; doi:10.3389/fimmu.2021.716289)
Supplement: Supplementary file 3 [file Table_2.docx]

| Species | Assembly | Isolate | Sequence platform | RefSeq accession | GenBank accession | Submitter | Chromosome | Reference chromosome |
| --- | --- | --- | --- | --- | --- | --- | --- | --- |
| Human  (*Homo sapiens)* | GRCH38.p13 | Unknown | Whole genome sequencing | GCF_000001405.39 | GCA_000001405.28 | Genome Reference Consortium | 19 | NC_000019.10 |
| Chimpanzee  (*Pan troglodytes)* | Clint_PTRv2 | Yerkes chimp pedigree #C0471 Clint | PacBio  Illumina  BioNano | GCF_002880755.1 | GCA_002880755.3 | University of Washington | 19 | NC_036898.1 |
| Bonobo  (*Pan paniscus*) | Mhudiblu_PPA_v0 | Mhudiblu carbone #601152 | PacBio RSII  Illumina | GCF_013052645.1 | GCA_013052645.1 | University of Washington | 19 | NC_048258.1 |
| Gorilla  (*Gorilla gorilla*) | Kamilah_GGO_v0 | Kamilah stud number 0661 | PacBio RSII  Illumina | GCF_008122165.1 | GCA_008122165.1 | University of Washington | 19 | NC_044621.1 |
| Orangutan  (*Pongo abelii*) | Susie_PABv2 | Susie | PacBio  Illumina NextSeq 500  BioNano | GCF_002880775.1 | GCA_002880775.3 | University of Washington | 19 | NC_036922.1 |
| Gibbon  (*Nomascus leucogenys*) | Asia_NLE_v1 | Asia | PacBio  Illumina | GCF_006542625.1 | GCA_006542625.1 | University of Washington | 10 | NC_044390.1 |
| Rhesus macaque (*Macaca mulatta*) | Mmul_10 | AG07107 | PacBio RSII | GCF_003339765.1 | GCA_003339765.3 | Genome Institute at Washington University School of Medicine | 19 | NC_041772.1 |
| Cynomolgus macaque  (*Macaca fascicularis*) | Macaca_fascicularis_5.0 | Unknown | Illumina HiSeq | GCF_000364345.1 | GCA_000364345.1 | University of Washington | 19 | NC_022290.1 |
| Common marmoset (*Callithrix jacchus*) | Callithrix_jacchus_cj1700_1.1 | Cj1700 | PacBio  Ilumina | GCF_009663435.1 | GCA_009663435.2 | McDonnell Genome Institute at Washington University | 22 | NC_048404.1 |

**Table S2: Genome information for each primate used in this overview**
