# Supplementary material for: The Genomic Organization of the LILR Region Remained Largely Conserved Throughout Primate Evolution: Implications for Health And Disease
Source: Front Immunol. 2021 Oct 19;12:716289. doi: 10.3389/fimmu.2021.716289 (PMC8562567; doi:10.3389/fimmu.2021.716289)
Supplement: Supplementary file 5 [file Table_4.docx]

**Table S4: Similarity comparison of KIR3DX1 and KIR3DX2 sequences in different primate species.**

For KIR3DX1, the chimpanzee sequence is used as reference, whereas rhesus macaque transcripts were used as reference for KIR3DX2. The similarities are considered for the coding region (Exons) and for the genomic sequence (gDNA), and shown in percentage. In KIR3DX2, the sequence is compared up to exon 5.

| **Chimpanzee *KIR3DX1* to other *KIR3DX1*** | **Exons** | **gDNA** |
| --- | --- | --- |
| Gorilla | 97.9 | 94.2 |
| Orangutan | 95.1 | 83.5 |
| Gibbon | 94.3 | 89.8 |
| Rhesus macaque | 90.8 | 76.6 |
| Cynomolgus macaque | 91.0 | 78.7 |
| Common marmoset | 68.4 | 59.3 |
|  |  |  |
| **Rhesus macaque *KIR3DX2* to other *KIR3DX2*** | **Exons** | **gDNA** |
| Gibbon | 73.1 | 35.9 |
| Cynomolgus macaque | 99.9 | 99.5 |
| Common marmoset | 80.4 | 57.5 |
|  |  |  |
| **Chimpanzee *KIR3DX1* to *KIR3DX2*** | **Exons** | **gDNA** |
| Gibbon | 59.4 | 40.6 |
| Rhesus macaque | 83.7 | 61.6 |
| Cynomolgus macaque | 83.8 | 61.6 |
| Common marmoset | 84.5 | 62.8 |
